# *Escherichia coli* Aggravates Calcium Oxalate Stone Formation via PPK1/Flagellin-Mediated Renal Oxidative Injury and Inflammation

**DOI:** 10.1155/2021/9949697

**Published:** 2021-07-13

**Authors:** Lingyue An, Weizhou Wu, Shujue Li, Yongchang Lai, Dong Chen, Zhican He, Zhenglin Chang, Peng Xu, Yapeng Huang, Min Lei, Zheng Jiang, Tao Zeng, Xinyuan Sun, Xuan Sun, Xiaolu Duan, Wenqi Wu

**Affiliations:** ^1^Department of Urology, The Second Affiliated Hospital, Guangzhou Medical University, Guangzhou, Guangdong 510260, China; ^2^Department of Urology, Minimally Invasive Surgery Center, Guangdong Key Laboratory of Urology, The First Affiliated Hospital of Guangzhou Medical University, Guangzhou 510230, China; ^3^Department of Urology, Shenzhen Shockwave Lithotripsy Research Institute, The Eighth Affiliated Hospital, Sun Yat-sen University, Shenzhen, Guangdong 518033, China

## Abstract

*Escherichia coli* (*E. coli*) is closely associated with the formation of kidney stones. However, the role of *E. coli* in CaOx stone formation is not well understood. We explored whether *E. coli* facilitate CaOx stone formation and its mechanism. Stone and urine cultures were reviewed from kidney stone formers. The ability of calcium oxalate monohydrate (COM) aggregation was detected to evaluate the influence of uropathogenic *E. coli*, then gel electrophoresis and nanoLC-MS/MS to detect the crystal-adhered protein. Flagellin (Flic) and polyphosphate kinase 1 (PPK1) were screened out following detection of their role on crystal aggregation, oxidative injury, and inflammation of HK-2 cell *in vitro*. By transurethral injection of wild-type, *Ppk1* mutant and *Flic* mutant strains of *E. coli* and intraperitoneally injected with glyoxylate in C57BL/6J female mice to establish an animal model. We found that *E. coli* was the most common bacterial species in patients with CaOx stone. It could enhance CaOx crystal aggregation both *in vitro* and *in vivo.* Flagellin was identified as the key molecules regulated by PPK1, and both of them could facilitate the crystal aggregation and mediated HK-2 cell oxidative injury and activated the inflammation-related NF-*κ*B/P38 signaling pathway. Wild-type strain of *E. coli* injection significantly increased CaOx deposition and enhanced oxidative injury and inflammation-related protein expression, and this effect could be reversed by *Ppk1* or *Flic* mutation. In conclusion, *E. coli* promotes CaOx stone formation via enhancing oxidative injury and inflammation regulated by the PPK1/flagellin, which activated NF-*κ*B/P38 pathways, providing new potential drug targets for the renal CaOx calculus precaution and treatment.

## 1. Introduction

Renal caliculus is a common and multifactorial disease affecting the population worldwide [1–3], which can be triggered by metabolic disorders, ethnic factors, oxidative injury, and urinary tract infections (UTIs). Approximately 80% of urinary stone contains calcium oxalate (CaOx), and the CaOx stone is the most common type, but the mechanism of its formation remains unknown [4, 5]. Infection stones, mainly composed of magnesium, ammonium, and phosphate, have been proven to be highly correlated with UTI [6]. Urease-producing bacteria (e.g., *Proteus* and *Klebsiella*) promote the formation of infection stones by changing the chemical environment of the urine [7]. Metabolic factors are usually considered to be the main etiology of CaOx stones. However, UTI is often associated with CaOx stones, and *Escherichia coli* is the most common bacterial species causing UTIs, which may be associated with the formation of kidney stones [8, 9]. In addition, oxidative damage in renal tubular epithelial cell is currently considered to be one of the pivotal triggers for the formation of CaOx stones [10–12]. This injury provides substances for heterogeneous nucleation of CaOx crystals and enhances their adhesion to renal epithelial cells and then promotes the process of crystallization [13]. Nevertheless, the specific roles and the pivotal potential mechanism of *E. coli* in epithelial cell oxidative injury and CaOx stone formation remain to be further investigated and elucidated.

*E. coli* can adhere to cells with the help of virulence factors secreted by itself, which are affected by inorganic polyphosphate (polyP) and its metabolic enzymes. PolyP, whose synthesis is encoded by polyphosphate kinase 1 (PPK1), is directly correlated with mobility, biofilm development, quorum sensing, and virulence in *E. coli* [14, 15]. PPK1 is a crucial enzyme in the biosynthesis and degradation of polyP, which was the first found in *E. coli* by Kornberg [16]. *Ppk1* gene mutant strains lack toxicity-related functions, like motility, quorum sensing, exopolymer formation, and surface attachment [17]. However, the exact role of PPK1 in *E. coli*-mediated CaOx stone formation has not yet been well defined.

Thus, we hypothesized that *E. coli* could induce renal inflammation and enhances oxidative injury and then promotes CaOx formation in the kidney. The PPK1 and its downstream protein are involved in the regulation of *E. coli*-related inflammation and oxidative injury. More importantly, a previously unknown mechanism of *E. coli* promoting renal CaOx stone formation has been revealed and the conclusion provides new potential drug targets for the renal CaOx calculus precaution and treatment.

## 2. Materials and Methods

### 2.1. Stone and Urine Sample Analysis

Data of kidney stone formers treated with one-stage percutaneous nephrolithotomy between September 2016 and September 2018 were retrospectively reviewed. Patient's materials comprising stone composition, midstream urine culture (UC), and stone culture (SC) were collected to analyze the microbial spectrum of uropathogens. All stone specimens for SC were collected during operation. Briefly, stone fragments were pulverized with a sterile grinder for subsequent infrared spectroscopy analysis after an aseptic collection, which use a sterile container filled with saline.

### 2.2. Cell Lines and Cell Cultures

HK-2 cells, which were produced in the American Type Culture Collection (ATCC; Manassas, VA, USA), were cultured in DMEM/F-12 supplemented with 10% FBS at 37°C in a humidified atmosphere of 95% air and 5% CO_2_.

### 2.3. Strains and Plasmids

Wild-type *E. coli* CFT073 (WT-CFT073) was purchased from ATCC (ATCC700928). DH5*α*, pKD46, DH5*α*, pKD4, and DH5*α* were preserved in our laboratory. All *E. coli* CFT073 strains were grown in terrific broth (TB) medium (select pepton 10 g/L, yeast extract 23.6 g/L, K_2_HPO_4_ 9.4 g/L, and KH_2_PO_4_ 2.2 g/L) at 37°C with shaking at 200 rpm overnight. The culture was harvested at mid-log phase (OD_600_ = 0.5) and centrifuged at 2,300 g for 5 min at 25°C. An equal volume of glycerol was mixed with the precipitated bacteria and stored at -80°C until needed. The remaining supernatant was sterilized through a filter (0.22 *μ*m pore size) for the following experiments.

### 2.4. Construction of UPEC PPK1 and Flagellin Mutant Strains

Based on the *E. coli* CFT073 *Ppk1* and *Flic* gene sequence (GenBank Accession No. NC_004431.1), primers H1-K1, H2-K2, H3-K3, and H4-K4 were synthesized to contain homologous arms. The sequences of the primers were as follows: PPK1-F: 5′-ttgctcgccataatatccaggcagtgtcccgtgaataaaacggagtaaaaGTGCGCTCTCCTGTTCCGAC-3′ and PPK1-R 5′-tgtagtcgtaaatcgccaactgcgcccgtactttgcggcgattgccgcggAGGTGGCACTTTTCGGGGAAATG-3′. External identification of the primers is as follows: F: 5′-cccaggaacacgctatttatcc-3′, R: 5′- catctaccacacgggctatgac-3′, I-F: 5′- tctgcgttactgccttgatgat-3′, I-R: 5′-tggtggaccgcgaaatcgcaac-3′, Flic-F: 5′-atcaacaagaaccagtctgcgctgtcgagttctatcgagcgtctgtcttGAGCTGCTTCGAAGTTCCTA-3′, Flic-R: 5′-atcaggcaatttggcgttgccgtcagtctcagttaatcaggttacggcgaCATATGAATATCCTCCTTAGTTCCTATTC-3′ and external identification of the primers: F: 5′-ccaacagcctctcgctgatcactc-3′, R: 5′-cacgttgctggcaaattaccattcatgttg-3′. The PPK1-F and PPK1-R were homologous to the marginal sequence of the *Ppk1* gene. The uppercase part was homologous to the sequence at both ends of the kanamycin resistance gene. PCR technology was used to amplify the targeting gene with kanamycin resistance and then purify it. Subsequently, *E. coli* CFT073/pKD46 electrotransformation competent cells were prepared, and the electrotransformation of target fragments was completed. Briefly, *E. coli* CFT073/pKD46 was cultured with 1 M L-arabinose (final concentration of 2 mM) to an OD600 of 0.5. Bacteria was precipitated and resuspended at low temperature with 10% glycerol. The purified target fragment was added to *E. coli* CFT073/pKD46 competent cells and then subjected to an electric shock (2.5 kV, 25 *μ*F, and 200 *Ω*). The transformed bacteria were grown in LB plates (containing 50 *μ*g/mL kanamycin) at 37°C overnight. After being genetically stable, the bacteria liquid is mixed with an equal volume of glycerol as the strain, stored at -80°C for subsequent experiments. Finally, the successfully constructed *Ppk1* and *Flic* mutants were named ∆PPK1-CFT073 and ∆Flic-CFT073, respectively.

### 2.5. COM Crystal Preparation

COM crystals were prepared basing on the published protocols [18]. The Tris buffers were made of Tris-Base and NaCl (pH 7.40, 90 mM Tris-Base, and 10 mM NaCl). Na_2_C_2_O_4_ (0.5 mM) and CaCl_2_ (5 mM) were diluted in the Tris buffer, respectively. The mixture was incubated 20 h at 20-25°C. The COM crystals were scraped under a microscope to observe and centrifuged (2000 g, 5 min). The methanol was removed after washing the COM crystals; then, COM crystals (per 1 mg dry weight) were air-dried and resuspended in terilized phosphate-buffered saline (PBS).

### 2.6. Comparison of the Aggregation Effect of Various *E. coli* Strains to COM

The effects of different bacterial strains on CaOx crystal aggregation were evaluated using 10^6^ CFU/mL. After coincubation with different strains of *E. coli* at 37°C for different times, CaOx crystal aggregation was evaluated as follows. The size of the COM crystals was measured from 100 random individual crystals. The average of each sample was calculated. The assembly of two or more individual COM crystals tightly combined was defined as crystal aggregation [19–21], which was counted and averaged from ten high-power fields. These experiments were performed in triplicates.

### 2.7. Comparison of the Aggregation Effect of Various *E. coli* Strains and Culture Medium to COM

To study the effect of secretion of extracellular molecules by *E. coli*, 100 *μ*L COM crystal buffer was added to 1 mL of medium sterilized by the filter as mentioned before and coincubated at 37°C. After 1 h, crystal images were taken using a phase-contrast inverted microscope (IX-71; Olympus, Tokyo, Japan) connected to a digital camera. The estimation of COM crystal size and COM crystal aggregation numbers were mentioned before. Control experiments were performed under the same conditions as PBS.

### 2.8. Screening of PPK1-Regulated Differential Molecules Secreted by *Δ*PPK1-CFT073 and WT-CFT073 Strains

After the WT-CFT073 and *Δ*PPK1-CFT073 strains and culture medium (sterile filtration as mentioned before) were incubated with COM (1 mg/mL) for 12 h, they were centrifuged for 10 min (1,200 rpm, 4°C), and then, the supernatant was discarded to obtain the incubated COM crystals. After washing the COM crystal three times with cold PBS, the RIPA lysate was added to isolate the adhesive proteins on the surface of the crystals. The proteins were denatured in road buffer (2% SDS, 10% glycerol, and 62.5 mmol/L Tris-HCl; pH 6.8). SDS-polyacrylamide gel electrophoresis (PAGE; 10% acrylamide) was used to separate protein. Then, the total protein was loaded in each lane. SDS-polyacrylamide gel was stained with colloidal Coomassie brilliant blue G-250 at room temperature for 18 h after proteins were separated. Coomassie Blue de-staining solution was used to wash for 12 h. The images were visualized using an automatic gel imaging system (Bio-Rad).

### 2.9. In-Gel Tryptic Digestion and Differential Protein Identification by nanoLC-MS/MS

The differential proteins were extracted from SDS-polyacrylamide gels and followed an in-gel tryptic digestion. EASY-nLC II (Bruker Daltonics; Bremen, Germany) was applied to isolate the digested sample. Mass spectra were deconvoluted using Data Analysis version 4.0 SP5 (Bruker Daltonics). MASCOT 2.3 software (Matrix Science, London, UK) was used to analyze the data of MS/MS spectra, and the protein database (uniprot_Escherichia_coli_4596_20170807.fasta) was analyzed on the UniProt Library (https://www.uniprot.org/).

### 2.10. Effect of Different Concentrations of Flagellin Antibody on COM Aggregation

Different concentrations of flagellin antibodies were preadded to the culture medium 30 min before it was incubated with COM (1 mg/mL). CaOx crystal aggregation was evaluated as previously described.

### 2.11. Crystal-Cell Adhesion Assay

HK-2 cells (10^6^ cells per dish) were seeded into 6 cm cell culture dishes until reaching 90% confluence (37°C, 5% CO_2_ incubator). Afterwards, the cells were infected with bacteria at a ratio of 10 bacteria per host cell for 1 h. 20 *μ*g/cm^2^ COM crystals was added to the medium and maintained statically for 5 min. PBS was used to remove the nonadherent crystals for three times. COM crystal adhesion were observed under an inverted microscope and images were taken, which were counted from at least 10 randomized high-power fields per well.

### 2.12. ROS Measurement

The process is described in Duan et al.'s study [22]. Briefly, intracellular ROS level was evaluated by dihydroethidium (DHE) fluorescence dye. The cells were pretreated with COM and different *E. coli* strains and then incubated with DHE (Sigma-Aldrich, USA) following the instruction. Then, we removed excess DHE and captured figures with a fluorescence microscope immediately. The final results were semiquantified by ImageJ software.

### 2.13. Animal Experiments

All experimental procedures attached to the guidelines of the National Institutes of Health Guide for the Care and Use of Laboratory Animals. The research was approved by Guangzhou Medical University Ethics Committee (GY2019-146). 42 C57BL/6J female mice (weight 15-21 g) were purchased from Guangdong Medical Laboratory Animal Center. All mice were raised in the Animal Experimental Center of Guangzhou Medical University; the rearing condition was controlled with a 12/12 hours light/dark cycle, a temperature of 22-26°C, and humidity of 40-70%. CaOx deposits in the kidneys of mice were established by injecting glyoxylate (glyoxylic acid, GA. 80 mg/kg, and 200 *μ*L) intraperitoneally (i.p.) for 7 days. On the first day, the mice were administered transurethral injection of bacteria to induce UTI under anesthesia (2-2.5% isoflurane induced anesthesia, followed by maintenance with 1.8-2%). The injection was repeated for 4 h as per the methods by Thai et al. [23] All mice were randomly divided into 6 groups (*n* = 8 per group): (1) normal group, (2) Ga i.p. only, (3) Ga+saline, (4) Ga i.p.+WT-CFT073, (5) Ga i.p.+△PPK1-CFT073, and (6) Ga i.p.+△Flic-CFT073. All mice were subjected to fasting for 12 h after the injecting the bacteria and then were provided food and water freely. Bilateral kidneys were removed under isoflurane anesthesia for histological examination and protein detection after 7 days.

### 2.14. Hematoxylin and Eosin (HE) and Pizzolato Staining to Detect the Kidney CaOx Crystals

Fixed kidney tissue samples were prepared paraffin-embedded sections (6 *μ*m), and hematoxylin and eosin solution was used to stain the tissues. COM crystals were detected by Pizzolato staining. The histological patterns of the kidneys were observed by HE. The CaOx crystal deposition of the kidney was confirmed by polarized light optical microphotography and tissue scanner. ImageJ software was used to calculate the crystal area.

### 2.15. Immunohistochemistry (IHC)

IHC followed a standardized process. Sections were incubated overnight at 4°C with anti-MCP1 (1 : 200 dilution, Abcam), anti-CD44 (1 : 100 dilution, Cell Signaling Technology), anti-p-P65 (1 : 200 dilution, Abcam), anti-p-P38 (1 : 200 dilution, Abcam) antibodies, anti-SOD1 (1 : 200 dilution, Proteintech), and anti-8-OHdG (1 : 200 dilution, Abcam). Subsequently, they were incubated with biotinylated horse anti-rabbit or anti-mouse antibody and avidin-biotin-peroxidase complex. The sections were dehydrated in ascending alcohol series. Finally, xylene was used to clear the sections. A polarizing microscope (CX31 Olympus, Tokyo, Japan) and tissue scanner (PathScope 4 s, DigiPath, NV, USA) were used to observe the sections. The protein expression of each antibody was calculated by ImageJ software.

### 2.16. Western Blot (WB)

The WB followed the previous research procedure [22]. The HK-2 cells pretreated with different bacteria were lysed in RIPA buffer. The cell proteins were extracted for quantitative denaturation, and then detected by WB. SDS-polyacrylamide gel used to separate proteins and then transferred to NC membrane (Millipore). Antibodies of CD44, p-P38, P38, p-P65, and P65 were immunoblotted with an NC membrane. HRP-conjugated anti-mouse and anti-rabbit IgG (sc-2005 and sc-2004, Santa Cruz) are secondary antibodies. Internal control was tubulin. ImageJ software was used to quantify and normalize processing.

### 2.17. Statistical Analysis

Mean ± SD was used to express all data. Statistical analyses were performed utilizing GraphPad Prism (version 8.0). *t*-tests and one-way ANOVA with LSD post hoc comparisons were used to complete data analysis. *p* < 0.05 have been viewed as statistically significant on the whole analyses.

## 3. Results

### 3.1. *E. coli* Is the most Common Bacterial Species in Urine and Stones of Patients with CaOx Stones

**Figure 1:**
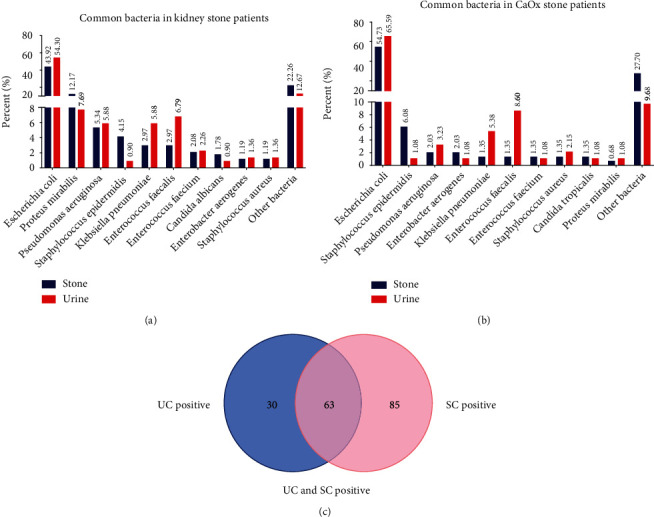
(a) Common bacteria in the urine and stone samples of patients with kidney stone. (b) Common bacteria in the urine and stone samples of patients with calcium oxalate stones. (c) The number of calcium oxalate stone patients with UC and SC both positive.

In total, 1055 patients were included. SC was positive in 337 (31.94%) patients, and UC was positive in 221 (20.95%) patients. The results of bacterial culture showed that among all stone types, *E. coli* accounted for 43.92% and 54.30% of all bacteria in stones and urine, respectively (Figure 1(a)). A total of 582 patients had CaOx stones (55.17%), SC was positive in 148 (25.43%) patients, and UC was positive in 93 (15.98%) patients. The percentages of *E. coli* were 54.73% and 65.59% in stones and urine, respectively (Figure 1(b)). In addition, among the 63 CaOx stone patients with both positive US and SC (Figure 1(c)), *E. coli* was detected in 48 (76.19%) UC and 47 (74.60%) SC. The bacterial spectrum of stones and urine from these patients indicated that *E. coli* dominated in the bacterial species of stones patients' urine and stone, regardless of the type of stone.

### 3.2. PPK1 Mediates WT-CFT073- and Its Culture Medium-Promoted COM Crystal Aggregation

**Figure 2:**
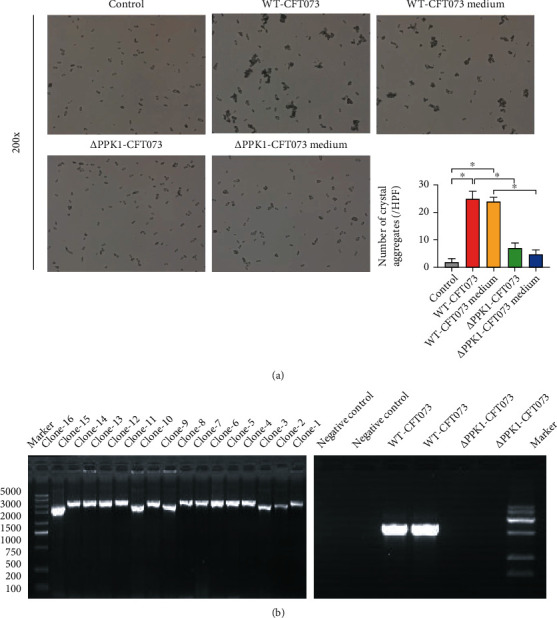
The aggregate effect of WT-CFT073, *Δ*PPK1-CFT073, and their medium on COM. (a) WT-CFT073 and its medium both promoted CaOx crystal aggregation (original magnification: ×200; the results are presented as the mean ± SEM. ^∗^*p* < 0.05 in comparison with the corresponding group). *Δ*PPK1-CFT073 and its medium reduced CaOx crystal aggregation. (b) *Ppk1* knockout UPEC strain was constructed and tested by PCR. Clone 1-16: the 16 clones randomly selected on the Kana resistance plate were numbered in sequence.

Compared to the control group, WT-CFT073 and its culture medium significantly promoted CaOx crystal aggregation. Interestingly, not only the WT-CFT073 strains but also its culture medium could promote COM aggregation (Figure 2(a)). These results indicated that some secretory virulence factors mediate the effect of *E. coli-*induced crystal aggregation.

The clone 2, 3, 9, 11, and 16 PCR fragments became smaller, in line with expectations, which were judged to be strain knockout *Ppk1* gene, and the *Ppk1* mutant (*Δ*PPK1-CFT073) was verified (Figure 2(b)). As shown in the crystal aggregation assay, compared to WT-CFT073, *Δ*PPK1-CFT073 and its culture medium induced weaker CaOx crystal aggregation (Figure 2(a)). No differences were found between control and *Δ*PPK1-CFT073, as well as the culture medium of *Δ*PPK1-CFT073. These results suggested that PPK1 is a vital factor for the effect of *E. coli* on crystal aggregation.

### 3.3. Differentially Expressed Proteins Involved in Crystal Aggregation and Secreted by WT-CFT073 Strain Were Regulated by PPK1

**Figure 3:**
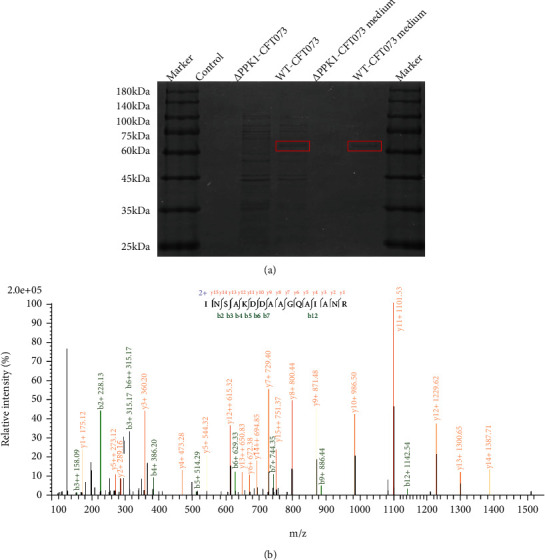
PPK1-regulated differential crystal aggregation relative molecules secreted by WT-CFT073 strain. (a) There existed differential protein bands with 60 kD and 75 kD between WT-CFT073 and *Δ*PPK1-CFT073. (b) The main differential protein is flagellin which analyzed by nanoLC-MS/MS.

After incubating the COM crystals with WT-CFT073, *Δ*PPK1-CFT073, and their respective cultures, the PPK1-regulated differential proteins related to aggregation were separated by gel electrophoresis, whereafter these proteins were identified by nanoLC-MS/MS. The differential protein bands were found to be markedly different at 60 kD and 75 kD (Figure 3(a)), and the results of Shotgun sequencing showed that the main differentially expressed protein was flagellin, which is the major component of bacterial motility organ flagellum (Figure 3(b)). These data indicated that flagellum is one of the key proteins involved in crystal aggregation regulated by PPK1.

### 3.4. Flagellin Mediated the Enhancing Effect of *E. coli* on COM Crystal Aggregation

**Figure 4:**
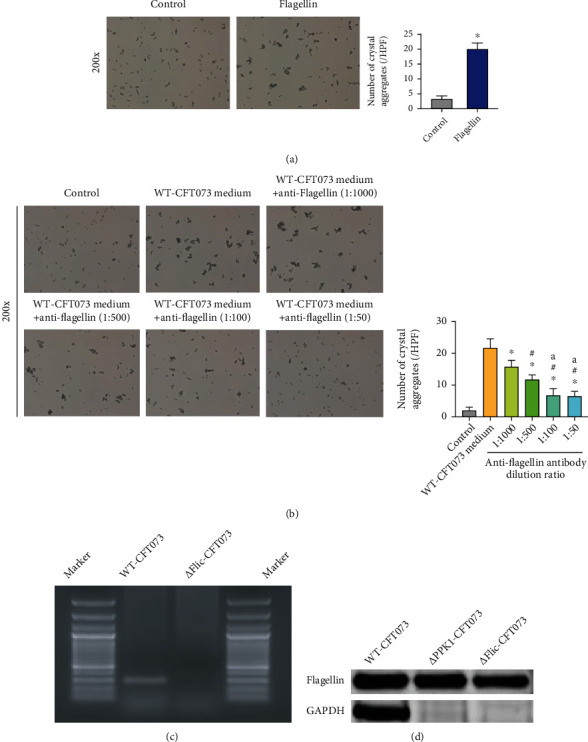
The aggregate effect of flagellin on COM and constructing flagellin mutant strain (*Δ*Flic-CFT073). (a) Flagellin showed a conspicuously aggregate ability on COM (^∗^*p* < 0.05 in comparison with the corresponding group; original magnification: ×200). (b) Anti-flagellin antibodies reduce the crystal aggregation of COM crystal compared with WT-CFT073 culture medium in a concentration dependent manner (^∗^^#a^*p* < 0.05, ^∗^compare to WT-CFT073 medium group, ^#^compare to 1: 1000 group, ^a^ compare to 1 : 500 group. Original magnification: ×200). (c) *Flic* knockout UPEC strain was constructed and tested by PCR. (d) Western blot showed the flagellin was decreased in *Δ*PPK1-CFT073 and *Δ*Flic-CFT073.

**Figure 5:**
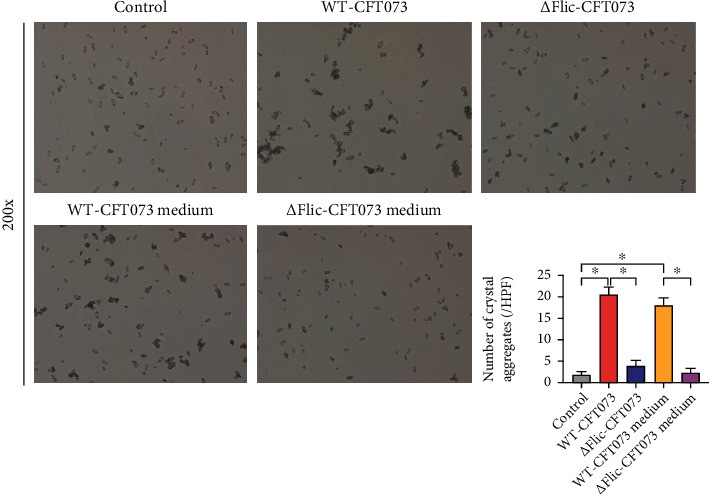
*Δ*Flic-CFT073 and its culture medium reduced CaOx crystal aggregation compared with WT-CFT073 (^∗^*p* < 0.05 in comparison with the corresponding group; original magnification: ×200).

The effect of flagellin on COM crystal aggregation was also examined. Flagellin treatment significantly increased crystal aggregation (Figure 4(a)). Pretreatment with anti-flagellin antibodies reversed the crystal aggregation induced by WT-CFT073 culture medium in a dose-dependent manner, and no differences were found between the 1 : 100 group and 1 : 50 group (Figure 4(b)). Furthermore, the *Δ*Flic-CFT073 strain had almost no flagellin expression (Figures 4(c) and 4(d)), and its culture medium induced less CaOx crystal aggregation compared with WT-CFT073. No differences were found between the control and *Δ*Flic-CFT073, as well as the culture medium of *Δ*Flic-CFT073 (Figure 5). These results indicated that the flagellum was indispensable for the role of *E. coli* in COM crystal aggregation.

### 3.5. PPK1/Flagellin Axis Was Essential for the Stimulatory Effect of *E. coli* on Crystal Adhesion

**Figure 6:**
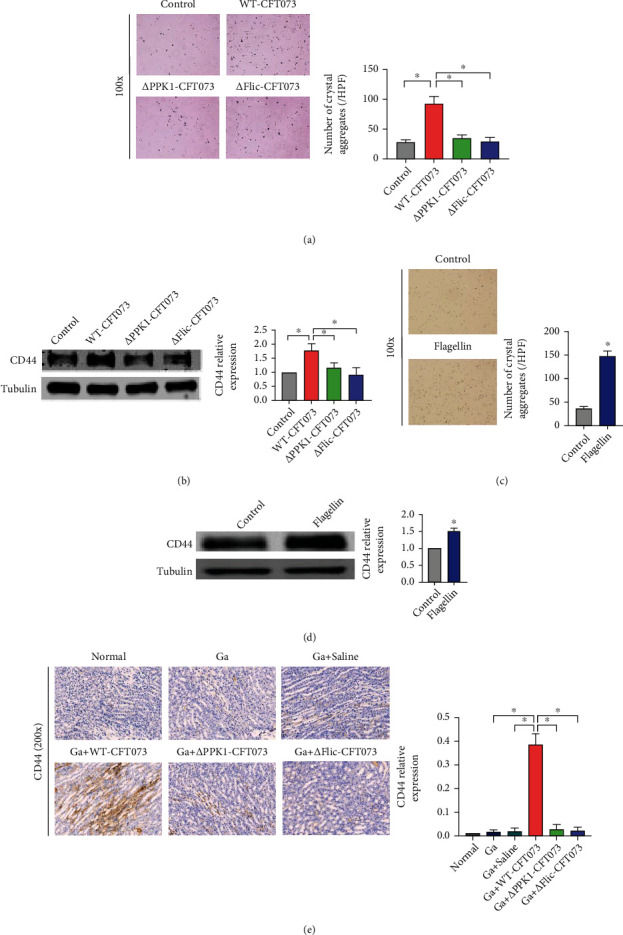
Flagellin is the key factor to the COM crystals adhesion *in vivo* and *in vitro*. (a) WT-CFT073-treated HK-2 showed higher adhesion COM (original magnification: ×100; ^∗^*p* < 0.05 in comparison with the WT-CFT073 group). (b) CD44 expression was decreased in COM injured HK-2 cells by *Δ*PPK1-CFT073 and *Δ*Flic-CFT073 treated (by Western blot, tubulin was used as an internal control. ^∗^*p* < 0.05 in comparison with the WT-CFT073 group). (c, d) The adhesion of COM crystals and the CD44 expression were increased by 100 ng/mL flagellin immediately treated (^∗^*p* < 0.05 in comparison with the control group). (e) CD44 expression was increased after transurethral injecting WT-CFT073 strain, but not *Δ*PPK1-CFT073 and *Δ*Flic-CFT073 strains (original magnification: ×200, ^∗^*p* < 0.05 in comparison with the Ga+WT-CFT07 group).

The amount of adhesive COM crystals in HK-2 cells and CD44 expression induced by *Δ*PPK1-CFT073 or *Δ*Flic-CFT073 strain were significantly reduced, compared with WT-CFT073 (Figures 6(a) and 6(b)). On the other hand, the adhesion of COM crystals and CD44 expression were increased by flagellin immediately after treatment (100 ng/mL flagellin-treated cells for 24 h) (Figures 6(c) and 6(d)). Using IHC, we found that an additional transurethral injection with WT-CFT073 led to higher CD44 expression than glyoxylic acid treatment alone. Particularly, the distribution of CD44 was located in the cytoplasm of renal tubular epithelial cells. In contrast, the additional transurethral injection with *Δ*PPK1-CFT073 or *Δ*Flic-CFT073 strain represented no significant effect on CD44 expression (Figure 6(e)). These *in vitro* and *in vivo* data suggest that the PPK1/flagellin axis mediates *E. coli-*induced CD44 overexpression and crystal adhesion to HK-2 cells.

### 3.6. *E. coli*-Induced CaOx Crystal Retention in Mouse Kidney Were PPK1 and Flagellin Dependent

**Figure 7:**
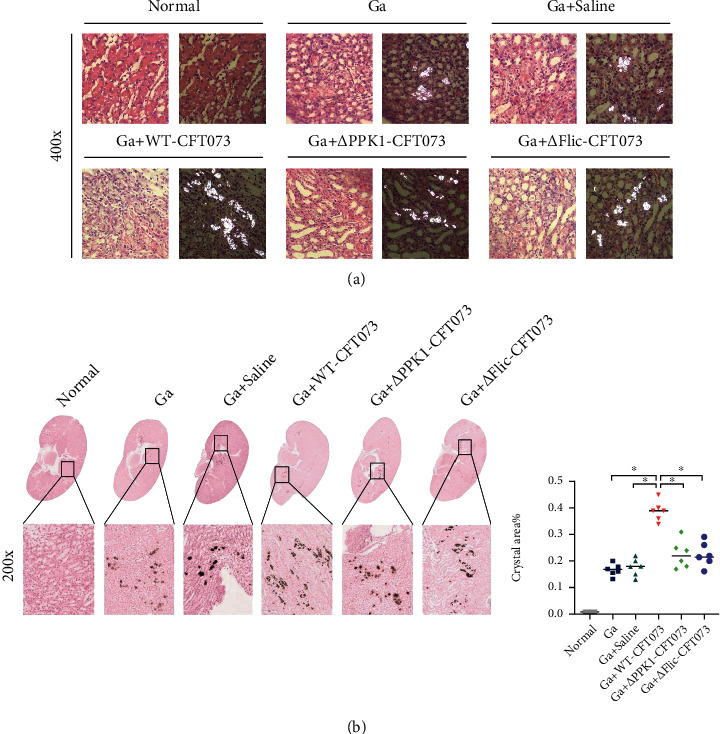
The CaOx deposition and histomorphology of mouse kidney after transurethral inject different bacteria strain. (a) HE and polarized light optical microphotography found that crystal depositions in Ga+WT-CFT073 were higher and larger; the renal tubules around crystals were obviously expanded and deformed (original magnification: ×400). (b) The Pizzolato staining (for detection of CaOx crystals) showed the area of CaOx crystals in the Ga+*Δ*PPK1-CFT073 and Ga+*Δ*Flic-CFT073 strain groups were decreased (original magnification: ×200, ^∗^*p* < 0.05 in comparison with the corresponding group). Ga: glyoxylic acid.

The amount of CaOx crystals retained in mouse kidneys exhibited the same trend as CD44 expression in the corresponding group. Using polarized light optical microphotography, the crystal retention in Ga+WT-CFT073 was found to be greater than that in the Ga+saline group. HE staining showed that the renal tubules around the crystals were obviously expanded and deformed (Figure 7(a)). In addition, the CaOx crystals were mainly located in renal cortex and medulla as observed by Pizzolato staining. In contrast to the Ga+WT-CFT073 group, the amount of CaOx crystals was less prominent in the Ga+*Δ*PPK1-CFT073 or Ga+*Δ*Flic-CFT073 group. No significant differences were observed among the Ga, Ga+saline, Ga+*Δ*PPK1-CFT073, and Ga+*Δ*Flic-CFT073 groups (Figure 7(b)). These data indicate that the PPK1/flagellin axis mediates *E. coli-*induced CaOx crystal retention *in vivo*.

### 3.7. *E. coli*-Induced Mouse Renal Oxidative Injury Regulated by PPK1/Flagellin

**Figure 8:**
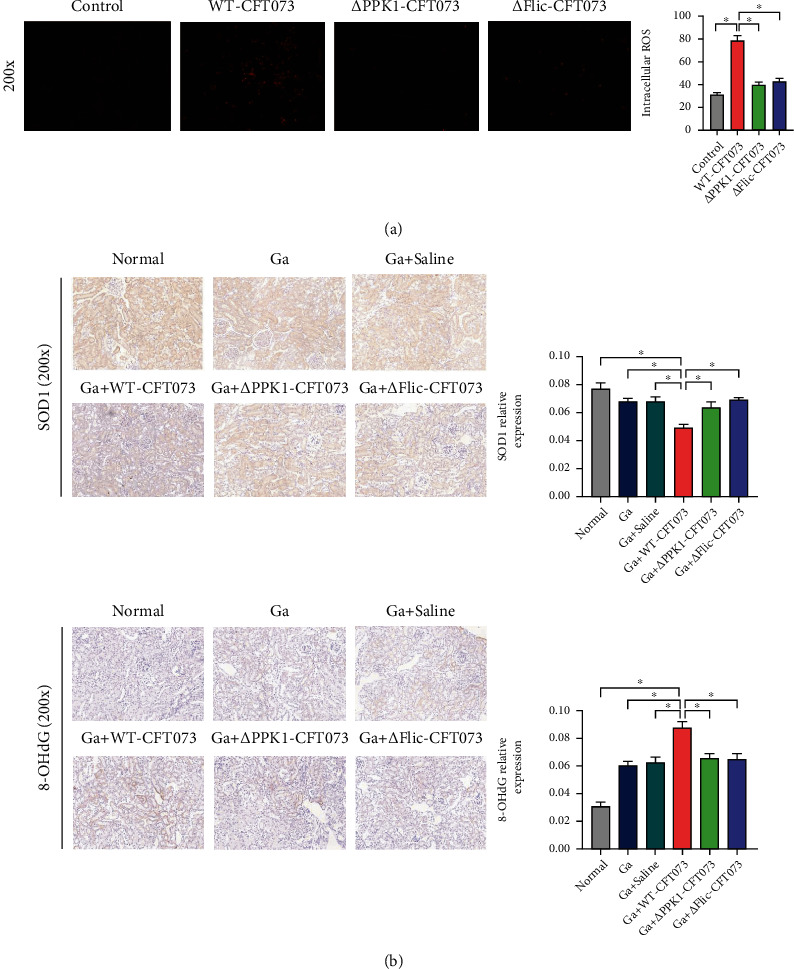
*E. coli*-mediated oxidative injury regulated by PPK1/flagellin. (a) HK-2 treated with WT-CFT073 were increased the intracellular ROS level (original magnification: ×200; ^∗^*p* < 0.05 in comparison with the WT-CFT073 group). (b) SOD1 was downregulated in the Ga+WT-CFT073 group, 8-OHdG was upregulated in the Ga+WT-CFT073 group, and both proteins reversed in *Δ*PPK1-CFT073 and *Δ*Flic-CFT073 (original magnification: ×200, ^∗^*p* < 0.05 in comparison with the WT-CFT073 group).

In the cell experiment, WT-CFT073 pretreatment clearly aggravated oxalate-induced ROS generation, which was not observed in the *Δ*PPK1-CFT073 and *Δ*Flic-CFT073 groups (Figure 8(a)). We then detected the oxidative injury markers in the mouse kidney. SOD1 expression was decreased clearly after transurethral injection of WT-CFT073 compared to *Δ*PPK1-CFT073 and *Δ*Flic-CFT073. In contrast, 8-OHdG protein (marker of oxidative damage in cellular DNA) expression increased in WT-CFT073. These increases were significantly attenuated by knockout *Ppk1* and *Flic* gene (Figure 8(b)). Together, these results suggested that wild-type *E. coli* accelerates oxalate-induced renal oxidative injury, which were reversed by knockout *Ppk1* and *Flic* genes.

### 3.8. PPK1/Flagellin-Mediated *E. coli*-Induced Mouse Renal Inflammation via Activation of NF-*κ*B/P38 Signaling Pathway

**Figure 9:**
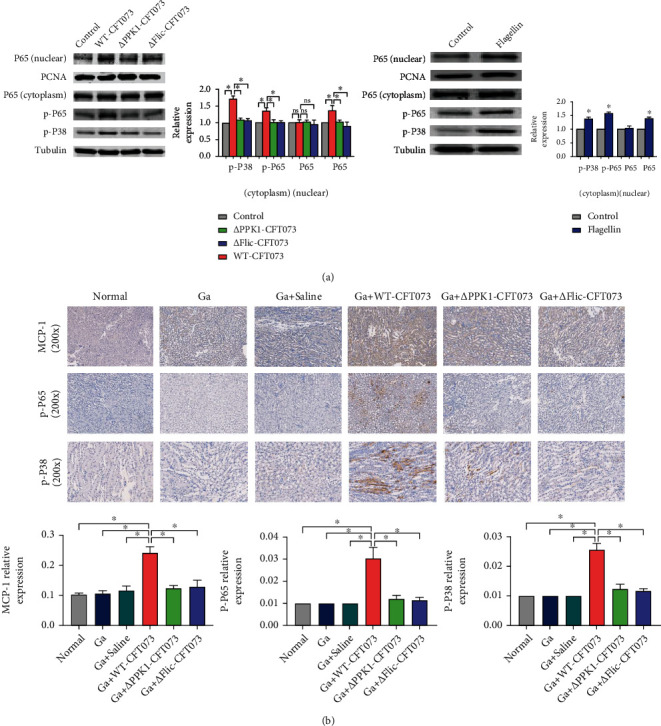
PPK1/flagellin may activate mouse renal inflammation via the NF-*κ*B/P38 signaling pathway. (a) HK-2 treated with WT-CFT073 and 100 ng/mL flagellin increased the intracellular and nuclear phosphorylation of P38 and P65 (by Western blot, tubulin was used as an internal control, ^∗^*p* < 0.05 in comparison with the WT-CFT073 group). (b) MCP-1, p-P65, and p-P38 were upregulated in the Ga+WT-CFT073 group, but not other groups. Phosphorylation of P65 and P38 were located in both the cytoplasm and nuclear of renal tubular epithelial cells (original magnification: ×200, ^∗^*p* < 0.05 in comparison with the WT-CFT073 group).

CaOx crystal deposition in the kidney is the primary driver of tubular epithelial injury, which induces localized inflammation in the kidney. In turn, renal inflammation is a key regulator of CaOx crystal deposition. Our results showed that stimulation with 100 ng/mL flagellin significantly increased intracellular phosphorylated P38 and P65, as well as nuclear P65 expression in HK-2 cells. Exposure to the WT-CFT073 strain also induced the same change, which was PPK1 and flagellin dependent. (Figure 9(a)).

In animal experiments, the expression of MCP-1, p-P65, and p-P38 was upregulated in the kidneys of mice injected with WT-CFT073. MCP-1 was mainly located in the cytoplasm of the renal epithelial cells. Weak expression of p-P65 and p-P38 was observed in the Ga+*Δ*PPK1-CFT073 and Ga+*Δ*Flic-CFT073 groups, compared with the Ga+WT-CFT073 group. Negative expression of MCP-1, p-P65, and p-P38 was observed in the normal, Ga, and Ga+saline groups (Figure 9(b)). These results indicate that *E. coli* could activate the mouse renal inflammatory NF-*κ*B/P38 signaling pathway, which is regulated by the PPK1/flagellin axis.

## 4. Discussion

Both *E. coli* and its medium promote the aggregation and adhesion of CaOx crystals in our research and that PPK1/flagellin plays a pivotal regulatory role in this process. *E. coli* enhances the renal oxidative injury and induces the renal inflammation via PPK1/flagellin expression, both of which further exacerbate the deposition of CaOx crystals in the kidney, at least, partly activating the NF-*κ*B/P38 signaling pathway.

As the most common type of urinary tract stones, CaOx stones have a high incidence worldwide and have a high chance of recurrence after treatment. Various studies have shown that bacteria in the urinary tract are closely associated with the formation of urinary tract stones. By altering the urine composition, urease-producing bacteria (e.g., *Proteus mirabilis*) could cause the formation of infection stones. The exosomes secreted by bacteria, including proteins, polysaccharides, and lipids, could also be involved in the formation of infection stones [5, 24]. In addition to infection stones, bacteria are commonly detected in metabolic stones. Approximately 40%-80% of patients with CaOx stones have concomitant UTIs, which are generally considered to be secondary to CaOx stones rather than a causative factor for its formation [25, 26]. Our study indicated that *E. coli* is the most common bacterial species detected both in CaOx stone former's urine and stone. Additionally, *E. coli* isolated from urine and stones in the same CaOx patient had similar characteristics, like consistent antimicrobial susceptibility profiles, genotyping, phylogenetic groups, virulence, and resistance genes [27]. Instead of the dead bacteria or their fragments, only living and intact *E. coli* bacteria promote nucleation and aggregation of CaOx crystals [20]. An *in vivo* study has confirmed that injection of *E. coli* into the urinary tract of mice causes renal inflammation and promotes crystal deposition [28]. Nevertheless, none of these studies have explored the key factors and specific mechanisms by which *E. coli* promotes CaOx calculus formation, in greater detail. Intriguingly, we confirmed that even in the absence of *E. coli*, the culture medium of wild-type *E. coli* promotes the aggregation of CaOx crystals, indicating that some regulatory molecules secreted by *E. coli* may be the key factors leading to CaOx crystal aggregation *in vitro*.

The pathogenicity of bacteria is closely associated with the virulence factors. Polyphosphate (polyP) is directly linked to bacterial function. It is an inorganic salt that participates in physiological processes. In addition to storing energy, polyP also acts a regulated role in phosphate storage, metal ions chelator, alkaline ion buffering, and DNA access [14, 15]. PPK1 which was first found in *E. coli* in 1956 demonstrated the ability to participate in biosynthesis and degradation of polyP [16]. In the bacterial extracellular matrix, PPK1 is highly associated with the secretion and function of various virulence factors in bacterial resistance and is unique to UPEC [29]. Our study showed a significantly decreased COM aggregation-inducing effect *in vitro* and *in vivo* after knockout of *Ppk1* in the UPEC strain. *Ppk1* is essential for bacteria to form biofilms, which promote stone growth and adhesion. Thus, as the biofilm-generating ability of △PPK1-CFT073 was greatly reduced, it resulted in weaker aggregation of the COM crystals. In addition, PPK1-deficient bacteria are more susceptible to environmental effects. The bacterial activity and resistance also decreased due to PPK1 deficiency. Therefore, it is reported that PPK1 may be a potential target for addressing the issue of bacterial resistance [30].

PPK1 is associated with multiple downstream factors that are secreted by bacteria. We found that flagellin is the most important crystal aggregation-related protein, associated with PPK1. It is the main component of the bacterial flagellum, which is a motor organ of bacteria that mediates swimming and mass movement, and assists in bacterial locomotion towards the host target site [31]. Flagellin is encoded by the *Flic* gene and folds into D0, D1, D2, and D3 domains in different spaces. The D1 domain could combine with the Toll-like receptor 5 in epithelial cells and monocytes, causing a strong inflammatory response [32]. *Flic* mutations lead to a significant reduction in the pathogenic capacity of bacteria [33]. In this study, flagellin was found to directly promote the aggregation of COM crystals, a finding that is also consistent with the study by Rattiyaporn et al. [34] Other components in *E. coli* also could aggregate COM crystals. For example, lipopolysaccharide- (LPS-) mediated negative charge on the bacterial surface may facilitate crystal aggregation, and the elongation factor Tu which is highly expressed in Outer membrane vesicles (OMVs) of UPEC also could promote the aggregation of COM crystals [34, 35]. However, their aggregation effect on COM crystals is significantly weaker than flagellin [34]. Our results validate the function of PPK1 in regulating flagellin expression and affecting the aggregation of COM crystals, indicating that flagellin is an essential factor in UPEC-induced COM crystal aggregation. In addition, the same phenomenon was observed in the mouse model. The formation of urinary tract stones involves biomineralization, and bacterial biofilm participates in this process [36]. Flagellin can accelerate the formation of the biofilm [30]. It might have the potential to alter the crystal surface charge and thus enable the *E. coli-*induced crystal aggregation [20]. From the points discussed above, it is clear that the role of flagellin in CaOx stone formation deserves more attention, and the specific roles of flagellin in biofilm formation and CaOx stone formation are expected to be revealed in the future.

High oxalate could promote crystal adhesion and oxidative injury of renal tubular epithelial cell, which are the pivotal processes in CaOx stone formation [22, 37, 38]. Since UPEC colonized in the urinary tract induces infections that can adhere to renal tissue and cause renal tubular epithelial cell damage via the action of matrix toxic force factors [39]. Our study confirmed that *E. coli-*induced crystal adhesion to HK-2 cells was reduced after *Ppk1* or *Flic* gene mutation. Overexpression of the adhesive capacity marker CD44 induced by *E. coli* was significantly weakened both *in vitro* and *in vivo*, if *Ppk1* or *Flic* was knocked out. Interestingly, treatment of HK-2 cells with flagellin increased CD44 expression and enhanced CaOx crystal adhesion. Additionally, the oxidative injury level also reversed in the △PPK1-CFT073 and △Flic-CFT073 groups. It means the ability of *E. coli* to promote CaOx crystal adhesion to renal tubular epithelial cells and to enhance oxidative injury is partly regulated by PPK1/flagellin, which may subsequently accelerate the formation of CaOx stones.

The present study also detected *E. coli-*induced differential marker protein associated with renal inflammation in mice. It has been reported that MCP-1, expressed in normal renal epithelial cells, is one of the key factors involved in the regulation of the inflammatory response [40]. It can be upregulated by stimulation of CaOx crystals and participates in CaOx-induced renal inflammation [41]. Our results showed that *E. coli-*induced inflammation was decreased after knockout of *Ppk1* and *Flic in vitro* and *in vivo*. It has been reported that the NF-*κ*B/P38 pathway-mediated inflammatory response is associated with CaOx crystal-induced renal tubular epithelial cell damage, which increased the expression of multiple surface proteins that mediate crystal adhesion like CD44, HA, and OPN, ultimately promoting the formation of CaOx stones [12]. It has been demonstrated that flagellin in *E. coli* activates the NF-*κ*B pathway to stimulate human epithelial cells to secrete inflammatory factors [42], which is consistent with our results. In addition, our study further indicated that the NF-*κ*B/P38 pathway activated by *E. coli* is partially regulated by the PPK1/flagellin axis. Weak inflammation was observed in the △PPK1-CFT073 and △Flic-CFT073 groups. This may be related to other virulence factors secreted by *E. coli*, which are less influenced by PPK1 (like LPS) [43, 44]. However, the results of us supported LPS may not a leading element in the aggregation of CaOx crystals, which is also consistent with Rattiyaporn's study [34].

Our study did not show whether inhibition of NF-*κ*B/P38- or PPK1-related inflammation pathways could reverse or alleviate the formation of stones, and the antioxidant activity regulated by PPK1/flagellin is unknown. Nonetheless, our research partly revealed the potential mechanism by which *E. coli* promotes CaOx stone formation via PPK1/flagellin, which may explain the results of Zhong et al.'s study [27]. In addition, whether the addition of anti-inflammatory drugs and antibiotics, together with anticalculus drugs, are helpful in reducing *E. coli*-induced CaOx stone formation, is also a vital question worth addressing in the application of CaOx treatment.

## 5. Conclusion

In summary, we found that *E. coli* is the most common bacterial species in stone and urine of patients with CaOx. We constructed *Ppk1* knockdown strains of *E. coli*, screened and identified the PPK1-regulated downstream crystal adhesion relative molecule flagellin by mass spectrometry. The dominant function of PPK1/flagellin in promoting CaOx crystal adhesion and aggregation was confirmed. In addition, we found that the ability of △PPK1-CFT073 and △Flic-CFT073 strains to induce renal oxidative injury and inflammation was weaker than that of the WT-CFT073 strain. Meanwhile, a previously unknown, specific mechanism, by which the PPK1/flagellin/NF-*κ*B/P38 inflammation pathway is activated by *E. coli* during the CaOx calculus formation, has been revealed. Taken together, this study not only highlights the significance of UTI in the lithogenous process of CaOx stones but also provides a new direction and potential drug targets for the CaOx calculus prevention and treatment.

## Data Availability

All the data supporting the results were shown in the paper can be applicable from the corresponding author.
